# Truncating de novo mutations in the Krüppel-type zinc-finger gene ZNF148 in patients with corpus callosum defects, developmental delay, short stature, and dysmorphisms

**DOI:** 10.1186/s13073-016-0386-9

**Published:** 2016-12-13

**Authors:** Servi J. C. Stevens, Anthonie J. van Essen, Conny M. A. van Ravenswaaij, Abdallah F. Elias, Jaclyn A. Haven, Stefan H. Lelieveld, Rolph Pfundt, Willy M. Nillesen, Helger G. Yntema, Kees van Roozendaal, Alexander P. Stegmann, Christian Gilissen, Han G. Brunner

**Affiliations:** 1 Department of Clinical Genetics, Maastricht University Medical Center (MUMC+), PO Box 5800, 6202 AZ Maastricht, The Netherlands; 2 Department of Genetics, University of Groningen, University Medical Center Groningen (UMCG), Groningen, The Netherlands; 3 Department of Medical Genetics, Shodair Children’s Hospital, Helena, MT USA; 4 Department of Genetics, Radboud University Medical Center (RUMC), Nijmegen, The Netherlands

**Keywords:** ZNF148, ZBP-89, Whole exome sequencing, Intellectual disability, Corpus callosum development, De novo mutations, Premature termination codon

## Abstract

**Background:**

Krüppel-type zinc finger genes (ZNF) constitute a large yet relatively poorly characterized gene family. ZNF genes encode proteins that recognize specific DNA motifs in gene promotors. They act as transcriptional co-activators or -repressors via interaction with chromatin remodeling proteins and other transcription factors. Only few ZNF genes are currently linked to human disorders and identification of ZNF gene-associated human diseases may help understand their function. Here we provide genetic, statistical, and clinical evidence to support association of ZNF148 with a new intellectual disability (ID) syndrome disorder.

**Methods:**

Routine diagnostic exome sequencing data were obtained from 2172 patients with ID and/or multiple congenital anomalies.

**Results:**

In a cohort of 2172 patient–parent trios referred for routine diagnostic whole exome sequencing for ID and/or multiple congenital anomalies (MCA) in the period 2012–2016, four patients were identified who carried de novo heterozygous nonsense or frameshift mutations in the ZNF148 gene. This was the only ZNF gene with recurrent truncating de novo mutations in this cohort. All mutations resulted in premature termination codons in the last exon of ZNF148. The number of the de novo truncating mutations in the ZNF148 gene was significantly enriched (*p* = 5.42 × 10^−3^). The newly described ZNF148-associated syndrome is characterized by underdevelopment of the corpus callosum, mild to moderate developmental delay and ID, variable microcephaly or mild macrocephaly, short stature, feeding problems, facial dysmorphisms, and cardiac and renal malformations.

**Conclusions:**

We propose ZNF148 as a gene involved in a newly described ID syndrome with a recurrent phenotype and postulate that the ZNF148 is a hitherto unrecognized but crucial transcription factor in the development of the corpus callosum. Our study illustrates the advantage of whole exome sequencing in a large cohort using a parent–offspring trio approach for identifying novel genes involved in rare human diseases.

## Background

The human genome contains over 400 Krüppel-type zinc finger (ZNF) genes. For many of these genes a biological function has not been elucidated thus far [1]. ZNF genes encode DNA-binding proteins, related to the archetypal *Drosophila* regulatory protein *Krüppel*, which recognizes specific DNA sequence motifs in gene promoters. They bind the major groove of the double helix by their C2H2 zinc finger domains, each consisting of a chain of two cysteines and two histidines that fold around and are stabilized by a single Zn^2+^ ion. ZNF genes may function as either transcriptional co-activators or repressors. They do so by promoting the binding of transcription factors to their cognate DNA recognition site or by recruitment of chromatin remodeling proteins such as histone deacetylases, methyltransferases, and demethylases [1, 2]. Most ZNF genes have been characterized using in vitro binding and reporter assays for identification of their target genes. By identifying ZNF genes involved in human disease, their broader role in ontogeny or physiological processes may be elucidated. Despite the extensive size of the gene family, the number of disease-linked ZNF genes remains relatively small. Here we identified truncating de novo mutations in the Zinc Finger Protein 148 (ZNF148; also known as ZBP-89 or ZFP148) from a large cohort of patient–parents trios referred for diagnostic exome sequencing in the period 2012–2016. These de novo mutations lead to syndromic intellectual disability (ID) with corpus callosum anomalies and short stature as shared features, accompanied by secondary variable microcephaly or mild macrocephaly, feeding problems, variable facial features, talipes, and malformations of the heart and kidneys.

## Methods

### Whole exome sequencing

For whole exome sequencing (WES), a parent–offspring trio approach was used as described by us previously [3, 4]. Exomes were sequenced using DNA isolated from blood according to standard procedures. Exome capture was done using the Agilent SureSelect v4 kit (Agilent, Santa Clara, CA, USA). Exome libraries were sequenced on an Illumina HiSeq instrument (Illumina, San Diego, CA, USA) with 101 bp paired-end reads at a median coverage of 75x. Sequence reads were aligned to the hg19 reference genome using BWA version 0.5.9-r16. Variants were subsequently called by the GATK unified genotyper, version 3.2-2 and annotated using a custom diagnostic annotation pipeline. De novo variants in index patients were called as described by de Ligt et al. [4]. Standard Sanger sequencing of patient and parental DNA was used for validation de novo variants identified in WES data.

### Statistical analyses

To assess whether de novo nonsense mutations in the ZNF148 gene occurred significantly more frequently in our ID/MCA patient cohort, we made use of the ZNF148-specific loss-of-function (LoF) mutation rates [5], exactly as we described previously in Lelieveld et al. [6]. The LoF rate was calculated by summing the individual ZNF148 specific de novo mutation rates for nonsense, splice site, and frame-shift variants. Null hypothesis testing of finding three nonsense or splice site mutations in the ZNF148 gene was done using a one-sided exact Poisson test based on a sample size of 2172 individuals with ID/MCA, representing 4344 alleles. Bonferroni correction for multiple gene testing was applied by multiplying the obtained *p* value by 19,280, i.e. the number of genes captured by the exome capture kit. To statistically assess whether de novo mutations clustered within the ZNF148 gene, random distribution of the mutations over the entire coding region of the gene (2382 bp) was simulated 10,000 times [6]. The mutual distance between these randomly applied mutations was calculated and statistically compared to the actual distances between the observed de novo mutations in our patients, which were present at c. positions 970, 1581, 1582, and 1792.

## Results

### Clinical reports

Patient 1 is a 6-year-old girl born at 35 + 4 weeks after an uncomplicated pregnancy who presented with respiratory insufficiency and severe feeding problems requiring gavage feeding. Birth measurements were normal. Currently, she has gastrointestinal dysmotility with delayed gastric passage and receives most of her caloric intake through a gastrostomy tube. Biochemical analysis of mitochondrial enzymes showed no abnormalities. Developmental milestones were reached late with independent walking and first words spoken at the age of 3 years. Her first teeth erupted after 3 years. She is hyperactive at times and she gets upset in crowded environments. WISCIII-III-NL (2.6–7.11 years) intelligence test at 5.6 years showed a total intelligence quotient (IQ) of 59 (95% confidence interval (CI) 54–71), verbal IQ of 72 (68–85), and performance IQ score of 63 (57–77) indicating a mild intellectual delay. She attends a school for children with ID. Cardiac ultrasound for evaluation of a systolic murmur revealed a structurally normal heart. Physical examination at 6 years revealed the following. Her speech was difficult to understand because of poor pronunciation. She currently has mild developmental delay, hyperopia (+6.5 dioptres), secondary mild microcephaly (−2.76 SD) and short stature (−2.29 SD), upslanted eyes, notched nares with visible columella, grooved philtrum, slightly prominent lower lip, pointed chin, and hypertrichosis of the arms and back. Cardiac ultrasound for evaluation of a systolic murmur revealed a structurally normal heart. Complaints of exertional chest pain remain unexplained. Magnetic resonance imaging (MRI) of her brain showed a thin hypoplastic corpus callosum.

Patient 2 was the first child of healthy unrelated parents. Prenatal ultrasound showed multiple congenital anomalies: agenesis of the corpus callosum, right hydronephrosis, left multicystic kidney, and bilateral pes equinovarus. Birth was by Caesarian section after 31 + 2 weeks gestation because of fetal bradycardia. Apgar scores were 2-4-7 after 1, 3, and 5 min. After delivery, he was apneic, pale, hypotonic, and bradycardic with a heart rate of 60/min. Renal ultrasound showed multicystic dysplastic right kidney and left hydronephrosis. Cardiac ultrasound showed open ductus arteriosus. Physical exam after birth and postmortem exam revealed short stature (–2.5 SD) and a number of dysmorphic features including a small fontanel, flat occiput, coarse square shaped face, broad nasal bridge, wide-set eyes with infraorbital creases, retrognathia, upturned nose, prominent columella, long smooth prominent philtrum, narrow palate, retrognathia with small chin, wide-spaced inverted nipples, 3 cm palpable liver, sacral dimple, flat buttocks, small male genitalia with inguinal testes, small hands and feet with dorsal furrowing of the skin, right helical notch, bilateral prominent crus helici, vertical groove in the left ear lobe, short neck, superfluous skin with hypertrichosis on face, uppers arms, legs, and back, and talipes equinovarus with deeply grooved skin of foot soles. Infant Respiratory Distress Syndrome (IRDS) developed with poor circulation and lactic acidosis persisting until death 6 days after birth. Postmortem investigation including brain MRI confirmed the renal abnormalities and corpus callosum agenesis as well as wide intracerebral ventricles in an otherwise normal brain.

Patient 3 is an 11-year-old girl, the second child of healthy Caucasian parents, born at 38 + 5 weeks. Prenatal ultrasound at 32 weeks of gestation showed symmetric cerebral ventriculomegaly especially of the posterior horns. At birth, she was hypotonic and hyporeactive with respiratory insufficiency with Apgar scores of 5-6-8 at 1, 5, and 10 min. Because of feeding problems, gavage was needed during the first 5 days. A capillary malformation on her forehead, nose, and upper lip faded in due time. She took her first independent steps at 2 years and said her first words at the age of 3 years. Axial hypotonia remained evident throughout childhood. Fast head growth in her first year led to secondary macrocephaly at 2 years, while short stature developed at 2 years in the presence of growth hormone deficiency for which growth hormone therapy was started at 3 years. She is hyperactive, shows compulsive behaviors, has frequent temper tantrums, and was diagnosed with PDD-NOS. WISCIII intelligence test at the age of 8 years showed a total IQ of 58, verbal IQ of 62, and performance IQ score of 69. Due to early breast development at the age of 8 years, Lucrin was given to prevent further development of puberty. Periodic palpitations and tachycardia at the age of 7 years remain unexplained after cardiologic examination including ECG and cardiac ultrasound. Physical examination at 7.2 years showed long face, high slightly prominent forehead, prominent occiput, mild macrocephaly (around +2.5 SD), curly hair, trident hairline, down slanting palpebral fissures, minimal epicanthic folds, slightly hooded eyelids, prominent columella, malocclusion of upper and lower incisors, pointed chin, genua valga, and pedes plani. MRI scan of the brain revealed corpus callosum agenesis. The phenotype was considered suggestive of Noonan syndrome. However, no mutation was found after screening of all known genes linked with Noonan, Cardio-Facio-Cutaneous (CFC), and Costello syndromes.

Patient 4 is a 7-year-old girl, the third child of a 30-year-old mother and a 37-year-old father. Abnormal prenatal ultrasound findings, including cystic kidneys and a cardiac defect in the fetus, prompted amniocentesis which showed a normal female karyotype and normal FISH for 22q11 deletion. After delivery at 36 weeks after induction, she was apneic at birth responding to positive pressure ventilation (PPV); APGAR scores 4-7-8 at 1, 5, and 10 min. Birth measurements were normal and bilateral clubfeet were noted. Coarctation of the aorta associated with mitral valve stenosis, overall consistent with hypoplastic left heart syndrome spectrum, were found on postnatal echocardiogram and initially managed with prostaglandin E1, followed by balloon dilation and aortic repair. Renal ultrasound showed a right-sided multicystic kidney. She developed feeding problems requiring tube feedings and growth restriction with normal growth hormone status and bone age. Her development has been globally delayed from early on and at the age of 6 years she had an expressive language disorder with very restricted vocabulary compared to relatively good receptive language skills. She is able to use sign language and assistive communication devices; she receives special education through the public health system. Her behavior is generally mellow, but she has social anxiety and becomes easily overstimulated. Ophthalmology and orthopedics are following her for hyperopia with mild bilateral optic nerve hypoplasia and bilateral clubfeet, respectively. She underwent repeated tympanostomy and bilateral tube placement for recurrent otitis media associated with conductive hearing loss. Physical features at the age of 7 years include severe microcephaly (–8.7 SD) and short stature (–5.11 SD) and bilateral clubfeet. Her distinct craniofacial gestalt is characterized by an oval-shaped head and face with mild bitemporal constriction, deep-set eyes with upslanting, short and narrow palpebral fissures, mild telecanthus, a prominent nose with low-hanging prominent columella, midface hypoplasia, prognathia, thin lips, highly arched palate, widely spaced teeth of abnormal shape, unusual ears with underfolded helices, prominent anthelix, and underdeveloped attached earlobes. Her neurological exam is non-focal. A computed tomography (CT) scan of the head was suggestive of partial dysgenesis of the corpus callosum. MRI of her brain was not performed.

**Fig. 1:**
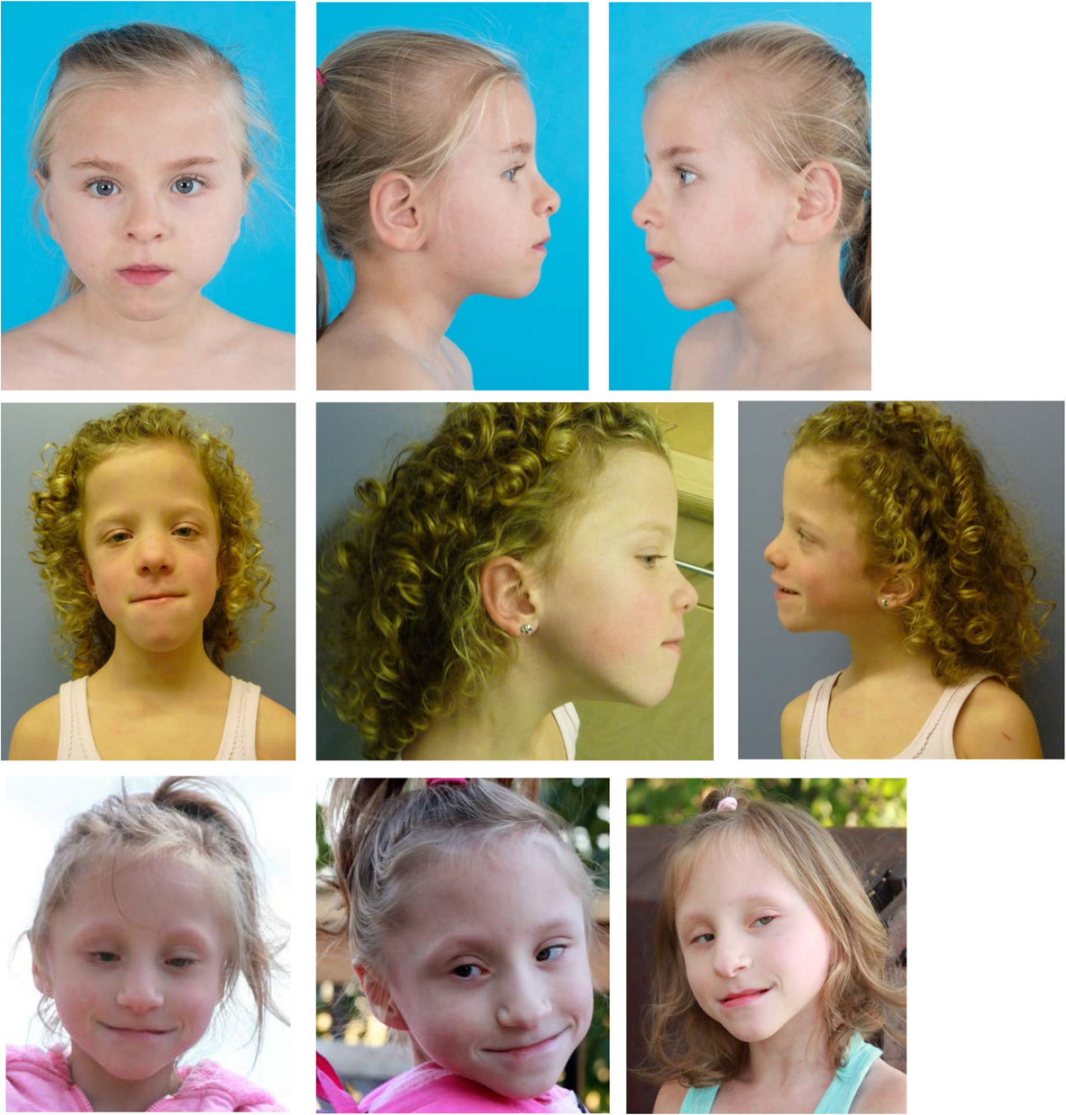
*Photographs* of (from *top* to *bottom*) patients 1, 3, and 4 with de novo truncating ZNF148 mutations. Note common features like triangular-shaped face with pointed chin and wide-set eyes

Clinical features of the four patients with ZNF148 mutations are summarized in Table 1. Clinical photographs are shown in Fig. 1.

**Table 1:** Clinical features of four patients with de novo mutations in the last exon of the ZNF148 gene

	Patient 1	Patient 2	Patient 3	Patient 4
Gender and current age	Girl, 6.7 years	Boy, died on postnatal day 6 after sudden bradycardia	Girl, 11.7 years	Girl, 7 years
ZNF148 mutation	c.1792A > T; p.Lys598*	c.1583dup; p.Ser529Glufs*2	c.970dup; p.Ser324Phefs*14	c.1581_1582insC; p.Lys528Glnfs*3
Additional variants	SART3 c.1526A > G; p.Asn509Ser TCERG1 c.2359G > A; p.Asp787Asn	None	PDCD4 c.1198C > G; p.Gln400Glu homozygous	COL3A1 c.3938A > G; p.K1313R; heterozygous, maternally inherited, MAF 0.26%; classified as VUS in ClinVar (RCV000181114.1)
Pregnancy	Uncomplicated, mother noted diminished fetal movements	Decelerative CTG just before birth	Uncomplicated	Renal cysts and heart defect on fetal ultrasound
Birth	Uneventful, 35 + 4 weeks	CS at 31 + 2 weeks, Apgar scores 2-4-7, not breathing, pale, hypotonic, and lactic acidosis postpartum	CS at 38 + 5 weeks, Apgar scores 5-6-8, hypotonic and hyporeactive, Continuous Positive Airway Pressure for respiratory insufficiency	Induced vaginal delivery at 36 + 0 weeks. Apgar scores 4-7-8. Apneic at delivery, responding to PPV. Started on PGE for known COA.
Birth weight	2.68 kg (–0.30 SD)	1.84 kg (+0.04 SD)	3.315 kg (+0.14 SD)	1.990 kg (–1.49 SD)
Birth length	47 cm (–0.37 SD)	39 cm (–2.62 SD)	49 cm (–0.50 SD)	42 cm (–2.55 SD)
Birth head circumference	32 cm (–0.75 SD)	29.2 cm (–0.37 SD)	35 cm (+0.49 SD)	28.5 cm (–3.2 SD)
Feeding problems	Severe, tube feeding need for sufficient caloric intake	Not applicable	Feeding problems during first week with 5 days of tube feeding	Feeding problems during neonatal period with tube feedings. Persistent FTT
Length	110.2 (–2.29 SD) at 6.6 years	40 cm (–2.5 SD) at 6 days	151.7 (+0.04 SD) at 11.3 years, catch up with growth hormone substitution therapy started at 3 years because of growth retardation (–2.48 at 2.7 years), and growth hormone deficiency	93.47 cm (–5.11 SD) at 6 years of age
Weight	17 kg (–1.09 SD for length) at 6.6 years	1.84 kg (+0.04 SD) at 6 days	38.4 kg (–0.33 SD for length) at 11.3 years	11.79 kg (0%ile, z-score –5.71) at 6 years of age
Head circumference	(–2.76 SD) at 6.5 years	29.2 cm (–0.37 SD) at 6 days	58.2 cm (+2.84 SD) at 11.3 years and fluctuating above and below +3 SD between 6 and 10 years	41 cm (–8.7 SD) at 6 years
Developmental milestones	Walked independently at 3 years. Spoke 3 years	Not applicable	Walked independently at 4 years and started talking > 3 years	Rolled over at 3 months. Crawled at 18 months. Walked independently at 4 years. Approximately 20 words
Cognition	WPPS1–III (2.6–3.1 years) at 3.7 years: TIQ 57 (95% CI 52–74) disharmonic profile: VIQ 72, PIQ 55. WPPSI-III-NL 2.6–7.11 years) TIQ 59 (95% CI 54–71), VIQ 72 (68–85), PIQ 63 (57–77) Mild intellectual delay	Not applicable	WISCIII at 8 years Total IQ 58, verbal IQ 62, perfomal IQ 69 Attends school for children with severe learning problems	No formal developmental assessment. Attends elementary school receiving special education through the public school system. Uses communication devices for expressive language. Receptive language seems good
Head	Triangular-shaped face with pointed chin	Coarse face, slight frontal bossing	Slight frontal bossing, triangular-shaped face with pointed chin	Oval-shaped face with mild bitemporal constriction
Hair	Normal blond straight head hair, hypertrichosis of arms and back	Hypertrichosis with lanugo hair on face	Curly hair	Fine hair
Eyes	Epicanthus, upslanted palpebral fissures Hyperopia +6.5 D	Slight right epicanthus, wide-set eyes, remarkable broad left eyebrow with long hairs	Wide-set, slight epicanthus, downslanting palpebral fissures	Mild telecanthus, upslanting, short and narrow palpebral fissures hyperopia, mild bilateral optic nerve hypoplasia
Nose	Full nasal tip, prominent columella	Long, smooth philtrum	Prominent columella	Prominent nose with low-hanging prominent columella
Philtrum	Deeply grooved	Smooth	Normal	Smooth
Mouth	Full lower lip	Normal	Wide-set points of upper vermilion	Wide mouth with thin upper vermillion border; highly arched palate, widely spaced teeth of abnormal shape
Ears	Prominent crus helicis of right ear	Large	Low-set and posteriorly rotated	Unusual shape with underfolded helix and prominent anthelix
Jaw	Pointed chin	Slight micrognathia, pointed chin	Pointed chin	Pointed chin with prognathia
Thorax	Normal	Wide-spaced inverted nipples	Normal	Normal
Limbs	Congenital trigger thumb, dysplastic nail of left hallux	Bilateral talipes equinovarus with deeply grooved foot soles	Pedes plani, slight genua valga	Bilateral talipes equinovarus
Genital	Normal	Undescended right testis	Normal	Normal
Puberty	No	No	Early breast development starting at 8 years	No
Epilepsy	No	+ (EEG burst suppression)	No	No
Brain	MRI brain: thin corpus callosum, slightly delayed myelination, suggestion of bilateral parieto-occiptalpolymicrogyria, periventricular hyperintensities	MRI brain: absent corpus callosum, wide lateral ventricles, bleeding	MRI brain: absent corpus callosum, colpocephaly	CT brain: suspected partial deficiency of the rostrum of the corpus callosum; non-specific foci in the left frontal and left occipital skull of unknown etiology. No MRI
Heart	Normal	Open ductus arteriosus, heavy heart – no evidence of cardiomyopathy	Normal	Coarctation of aorta, mitral valve stenosis
Kidneys	Renal ultrasound not done yet	Multicystic dysplastic left kidney, pyelectasia/ hydronephrosis of right kidney	Normal renal ultrasound	Multicystic dysplastic right kidney. History of multiple urinary tract infections. Normal voiding cystourethrogram
Endocrine	Not investigated	Not investigated	Early signs of puberty, growth hormone deficiency	Normal bone age and growth hormone status
Other	Recurrent upper airway infections, delayed intestinal mobility problems, late first tooth eruption > 3 years	Short neck	Frequent rhinitis in the first year	Frequent otitis media status post tympanostomy and tube placement

### Genomic analyses

**Fig. 2:**
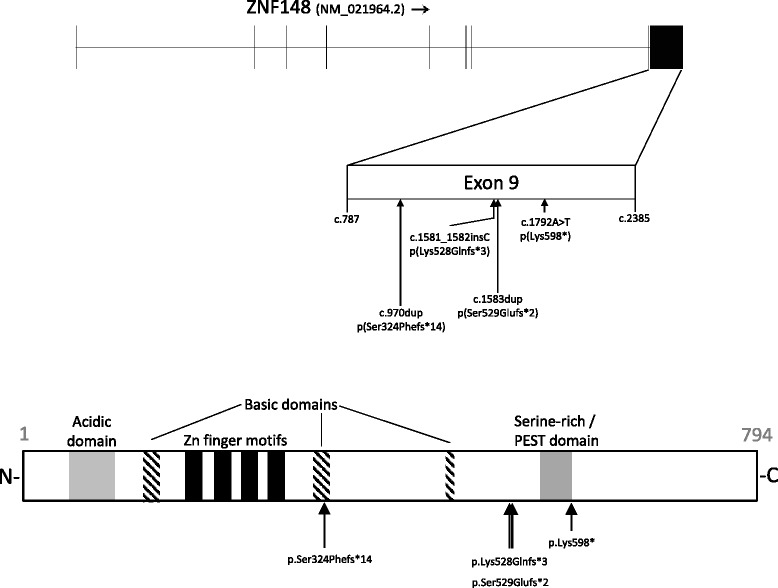
*Top*: Structure of the ZNF148 gene (NM_021964.2) located in chromosome band 3q21.2 and the location of the de novo mutations in the last exon (exon 9). *Bottom*: ZNF148 protein structure. The protein consists of 794 amino acids (AA). Structural domains in the ZNF148 protein include the acid domain (AA 54-99), three basic domains (AA 129-153, 313-335, and 470-485), four adjacent ZNFs (AA 173-278), a serine-rich / proline, glutamic acid, serine, threonine (PEST)-rich domain, and a C-terminal activation domain that interacts with other proteins (figure based on Merchant et al. [7])

During routine diagnostic WES in the Radboud University Medical Center (RUMC) in Nijmegen during the period 2012–2015 we identified patients 1–3, out of a total of 2172 WES trio analyses of patients with ID and/or MCA and both their parents. The three patients had heterozygous truncating de novo mutations in ZNF148, the only ZNF gene in which such mutations occurred recurrently in our cohort. Patient 4 was identified via GeneMatcher [7]. All four mutations were located in the last exon (exon 9) of the ZNF148 gene (reference sequence NM_021964.2) and were as follows: patient 1: c.1792A > T (p.Lys598*); patient 2: c.1583dup (p.Ser529Glufs*2); patient 3: c.970dup (p.Ser324Phefs*14); and patient 4: c.1581_182insC (p.Lys528Glnfs*3). These four de novo mutations all yield a premature termination codon (PTC) in the ZNF148 transcript, respectively, 458, 265, 197, and 265 codons upstream of the canonical wild-type termination codon (Fig. 2). Variants in additional genes were detected in three patients. Patient 1 had heterozygous *de novo* missense mutations of unknown significance in the TCERG1 gene (NM_006706.3:c.2359G > A; p.Asp787Asn) and in the SART3 gene (NM_014706.3:c.1526A > G; p.Asn509Ser), neither of which can be related to the clinical phenotype currently. Mutations in these two genes have not been described thus far and we did not observe other patients in our cohort harboring de novo mutations in these genes. Patient 3 showed a likely benign, homozygous missense variant in the PDCD4 gene (NM_014456.4:c.1198C > G; p.Gln400Glu), which has low heterozygous frequency in ExAC of 0.02% in Europeans [8] and which is also reported homozygously once in this database. In patient 4, a maternally inherited heterozygous missense variant in COL3A1 (NM_000090.3 c.3938A > G; p.Lys1313Arg), was found. This variant has been classified as variant of uncertain significance (VUS) in ClinVar as variant RCV000181114.1 [9]. However, neither the patient nor his mother or other individuals on the maternal side of the family have clinical symptoms suggestive of vascular Ehlers-Danlos syndrome (EDS type IV), the only syndrome known to be caused by pathogenic mutations in COL3A1. Moreover, this COL3A1 variant has a MAF of 0.26% in Europeans in the Exome Variant Server [10].

Next, we calculated the statistical probability of the number of de novo nonsense and frameshift mutations in our entire cohort of 2172 WES parent–offspring trios sequenced at RUMC. There was a significant increased number of such mutations in the ZNF148 gene in our ID/MCA cohort (*p* = 5.42 × 10^−3^). The distribution of the four mutations was not random over the coding region of the ZNF148 gene, but the mutations were significantly clustered (*p* = 0.033).

## Discussion

We detected de novo mutations in the last exon of ZNF148 in four patients and show significant enrichment of such mutations in our cohort of 2172 parent–offspring trios analyzed by WES. The core features in these four individuals with ZNF148-associated syndrome are: underdevelopment of the corpus callosum; mild to moderate developmental delay and ID; variable microcephaly or mild macrocephaly; short stature; feeding problems; facial dysmorphisms including wide-set eyes; low columella and pointed chin; and cardiac and renal malformations. But there were also noticeable differences between these four individuals. The clinical course in patient 1 is noteworthy for persisting feeding problems necessitating feeding by gastrostomy until now. Patient 2 had a coarse appearance and died from cardiac and pulmonary insufficiency on day 6. Patient 3 had mild secondary macrocephaly and developed secondary growth retardation with growth hormone deficiency and showed catch-up growth to normal height with GH replacement therapy. The phenotype of patient 3 was very suggestive of Noonan syndrome but testing showed no mutation in all genes associated with Noonan, CFC, and Costello syndrome. Patient 4 has very short stature and had severe microcephaly from birth. Her facial features were reminiscent of Floating Harbor syndrome (FHS).

The absence of mutations leading to PTCs in the ZNF148 gene in the general population, their significant overrepresentation in our cohort, and ZNF148 constraint metrics, i.e. “Probability of Loss-of-function Intolerance” (pLI) = 0.93 as described [8] and absence of nonsense or frameshift mutations in >100,000 alleles in ExAC [8] and a high rank haploinsuffiency (HI) score of 10.07 [11], strongly indicate that this gene is highly intolerant to truncating mutations. These calculations in combination with the phenotypical overlap in the identified patients strongly support causality for the de novo PTC mutations in ZNF148.

ZNF148 encodes a Krüppel-type zinc finger protein, with four C2H2 zinc finger motifs that bind similar DNA sequence elements in different gene promoters [12]. A BLASTP search [13] showed no apparent homology to other ZNF genes and the gene is relatively poorly characterized with regard to tissue-specific expression. However, ZNF148 mRNA is ubiquitously transcribed throughout numerous anatomical structures of the developing human fetal brain with highest expression in most regions until 16 weeks post conception [14, 15]. Unfortunately, no data are available on the gene’s expression in the developing corpus callosum but based on the patients’ phenotypes this is highly expectable. Homozygous ZNF148 knock-out mice are not viable while heterozygous mice show no consistency in observed phenotype [16]. Besides its four ZNF motifs, the protein contains an N-terminal acidic domain, three basic domains, a serine-rich/proline, glutamic acid, serine, threonine (PEST)-rich domain, and a C-terminal activation domain (Fig. 2 and [12]).

Over 30 target genes of ZNF148 have been identified to date [16]. A number of these target genes can be hypothetically linked to the clinical phenotype observed in the patients. For example, ZNF148 directly activates promoter activity of the growth hormone (GH) receptor [17, 18] and altered ZNF148 dosage or altered protein function may therefore be related to the short stature observed (Table 1). Likewise, in vitro reporter assays showed that ZNF148 is a key regulator of PKD1 and PKD2 gene promoters. The kidney abnormalities observed in patients 2 and 4 may therefore be related to aberrant ZNF148-driven PKD gene transcription [19]. The phenotype of patient 4 resembles FHS, which has some similarities with Rubinstein-Taybi syndrome (RTS) such as short stature, prominent columella, and renal and cardiac anomalies [20]. While our patients have a prominent columella and some have heart defects, the overall resemblance is still limited. It is notable though that ZNF148 binds the RTS-related EP300 protein, indicating that shared pathways may be affected in our patients and RTS patients. These pathways regulated by EP300-ZNF148 interactions are likely involved in chromatin remodeling by histon deacetylation via complexing with, for example, HDAC1 or HDAC3 [21–23].

All patients harboring ZNF148 mutations had maldevelopment of the corpus callosum (MCC), which can be related to abnormal neuronal proliferation or migration, abnormal telencephalic midline patterning, or abnormal axonal growth or guidance (reviewed in [24]). Although over 50 genes have currently been linked to MCC [24], none of these has clear overlap with ZNF148 with regard to biological function or the cellular pathways they are involved in. In addition, the clinical phenotype of our patients is different from known syndromes in which MCC is a feature [24]. We thus postulate ZNF148 as novel genetic factor required for corpus callosum development.

As all four de novo PTC mutations occurred in the last exon of the ZNF148 gene, they most probably do not to lead to nonsense-mediated RNA decay (NMD), but rather are likely to yield a truncated protein [25]. This pattern of “last exon de novo PTC mutations” is also observed for several other genes, where pathogenic PTCs only appear to be located in the last exon (occasionally with rare exceptions in the penultimate exon) or where last exon mutations give a distinct clinical phenotype, e.g. SOX10, EZH2, NOTCH3, KAT6A, and ASXL1 [26–30]. Although all four mutations in ZNF148 are distal to its C2H2 zinc finger domains, they truncate the protein in such a way that it is missing the larger part of the C-terminal activation domain (Fig. 2). These truncated proteins would be expected to have altered biological function, e.g. inability to interact with components of transcription-initiator or transcription-repressor complexes (LoF), to have a dominant negative effect or to have a “gain-of-function (GoF)” effect. The ExAC database currently lists two variants that may lead to NMD, both of which are located at the canonical splice acceptor site of intron 5. Still, it is difficult to draw conclusions on whether NMD of ZNF148 transcript is “benign,” because of: (1) the small number of individuals in ExAc (i.e. only two LoF mutations per ~110,000 alleles); (2) the lack of phenotypic data for these two individuals and the fact that corpus callosum abnormalities may have subtle clinical manifestation; and (3) the fact the splice effect of these two variants has only been predicted by in silico tools. No deletions or other genetic aberrations of ZNF148 have thus far been reported in literature, while the DECIPHER database [31] does currently not report single gene copy number variants for ZNF148. It remains therefore elusive whether HI of the ZNF148 gene can lead to a clinical phenotype and whether that phenotype would be similar to that associated with protein truncation, the likely mechanism in our patients. Thus, a rationale exists for further studies that investigate by which molecular mechanisms and molecular interactions the ZNF148 protein is involved in the genesis of clinical phenotypes, in particular corpus callosum abnormalities.

## Conclusion

Based on the de novo ZNF148 gene mutation rate in our patients, the nature of these mutations (i.e. de novo and truncating), the location of the observed mutations in the last exon, and the patients’ overlapping clinical phenotypes, we provide evidence that de novo truncating ZNF148 mutations cause a syndrome characterized by ID, short stature, aberrant head size (from microcephaly to mild macrocephaly), feeding problems, variable facial characteristics including telecanthus/wide nasal bridge, low columella and pointed chin, cardiac and renal malformations, and talipes. We furthermore postulate that the ZNF148 protein is a hitherto unrecognized but crucial transcription factor in the development of the corpus callosum. Our study illustrates the advantage of WES in a large cohort using a parent–offspring trio approach for identifying novel genes involved in rare human diseases, based on recurrence of mutations and clinical and statistical evidence.
